# Trends in body mass between finalist teams in the Japanese collegiate rugby union championship: a 15-year analysis

**DOI:** 10.3389/fspor.2025.1496093

**Published:** 2025-02-28

**Authors:** Hiroyuki Sagayama, Shogo Yamasaki, Akiko Uchizawa, Yuki Ahagon, Tatsuya Shimasaki

**Affiliations:** ^1^ Institute of Health and Sport Sciences, University of Tsukuba, Ibaraki, Japan; ^2^ Advanced Research Initiative for Human High Performance (ARIHHP), University of Tsukuba, Ibaraki, Japan; ^3^ Graduate School of Comprehensive Human Sciences, University of Tsukuba, Ibaraki, Japan

**Keywords:** body mass, rugby union, match outcomes, university athletes, position-specific analysis, nutritional support

## Abstract

This study examined the relationship between body mass and match outcomes among university rugby players in a Japanese collegiate rugby union championship over a 15-year period (2008–2022). Using data from 680 players across 30 finalist teams, we analyzed trends in body mass by playing position and their impact on match outcomes. No interaction was observed for body mass in matches over 15 years for all registered players (interaction [year × match], *p* = 0.85). Although no significant annual increase in body mass was observed across the period, the winning teams consistently had heavier players, with an average difference of 2.2 kg. The forwards of the winning teams were, on average, 3.6 kg heavier, and their reserve players for forward positions were 4.2 kg heavier than those of the losing teams. These findings emphasize that body mass is a crucial factor in determining success at university level, particularly in forward positions. This study highlights the need for further investigation of the role of body composition, physical assessments, and game-related factors to better understand the determinants of rugby performance.

## Introduction

Rugby is a sport in which competitors aim to secure possession of the ball and outsmart and overpower their opponents to cross the try line (1). Body size is a substantial advantage in this sport (2). During the scrum, eight forwards from each team push against one another to gain possession of the ball (3). In this scenario, larger players generate greater force (4). Although most individuals struggle to increase their maximum running speed and acceleration (5), they could change their body mass (4). Typically, forward players are larger than backs players because forward players are more involved in high-impact contact plays such as scrums (6). It is well known that the body mass of forwards positively impacts match performance (1, 7). Rugby players, particularly forwards, intentionally acquire more body mass during the off-season to improve their performance and endure high-intensity contact (8–10). Quarrie and Wilson (2000) highlighted that increased body mass correlates with better performance in collision-based tasks (11). Similarly, Ross et al. (2014) demonstrated that elite rugby forwards with greater body mass exhibit superior effectiveness in scrummaging and mauling (12). Woodhouse et al. (2023) further emphasized that greater body mass in rugby players is associated with enhanced physical dominance in matches (7). However, the relationship between body mass and matching outcomes remains unclear. Furthermore, improvements in diet and nutritional support in recent decades have led to an increase in male body mass globally (13), and it is anticipated that young athletes also benefit from these developments (14, 15). This phenomenon has been observed particularly in elite rugby players (4) whose body mass is thought to have increased in recent years. Based on recent improvements in growth and nutritional support (16), we hypothesized that the body mass of rugby players would significantly increase, and that weight would affect match outcomes.

This study aimed to clarify the relationship between the body mass of university rugby players and their match outcomes in finals, as well as the trends in body mass over the past 15 years.

## Materials and methods

The data for this study were obtained from open data sources (17), with confirmation from the Japan Rugby Football Union regarding appropriate use. Data have been made public since 2008 and used for the analysis of finalist championship teams from 2008 to 2022. The data included information on 22 registered players per team from 2008 to 2012, after which 23 registered players were included. Hence, the analysis was conducted using data from 680 individuals from 30 teams. The registered players were designated as follows: #1–#8 as forward players and #9–#15 as backs players. Furthermore, registered players #16 to #18 were defined as reserve A and #19 to #23 as reserve B.

### Statistical analyses

Tests were conducted to ensure that the assumptions underlying the variables were satisfied. The Kolmogorov–Smirnov test was used to assess normality and Levene's test was used to evaluate the homogeneity of variance. We subsequently performed a two-way analysis of variance (ANOVA) to examine the interaction between the variables of year (2008–2022) and match (win and lose). For match analysis, we used an unpaired t-test to compare winners and losers. Effect sizes were calculated using Cohen's d for t-tests and partial eta squared (*η*^2^) for ANOVA with the following thresholds: small (d = 0.2, partial *η*^2^ ≈ 0.01), medium (d = 0.5, partial *η*^2^ ≈ 0.06), and large (d = 0.8, partial *η*^2^ ≥ 0.14) (18–20). All data are reported as mean ± standard deviation. The level of statistical significance was set at *p* < 0.05. Statistical analyses were performed using GraphPad Prism 10.2.3 (GraphPad Prism Software Inc., La Jolla, CA, USA) and SPSS software (version 29.0; IBM Corp., Armonk, NY, USA).

## Results

**Figure 1:**
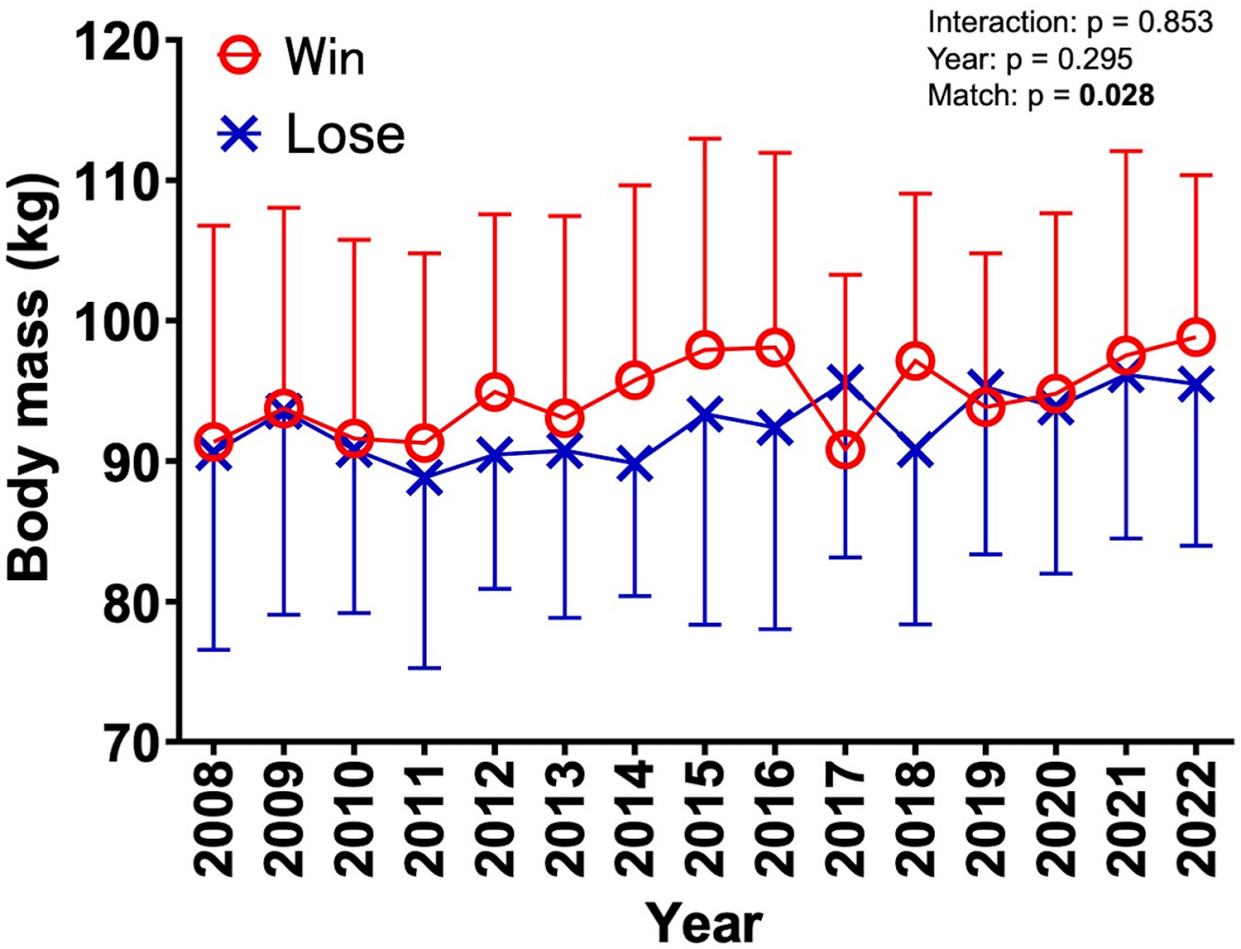
Yearly trends in body mass in relation to match outcomes (2008–2022). The mean body mass (kg) of the winning teams is represented by red circles (○), and the mean body mass of the losing teams is indicated by blue crosses (✕). The error bars represent the standard deviations for each year.

**Figure 2:**
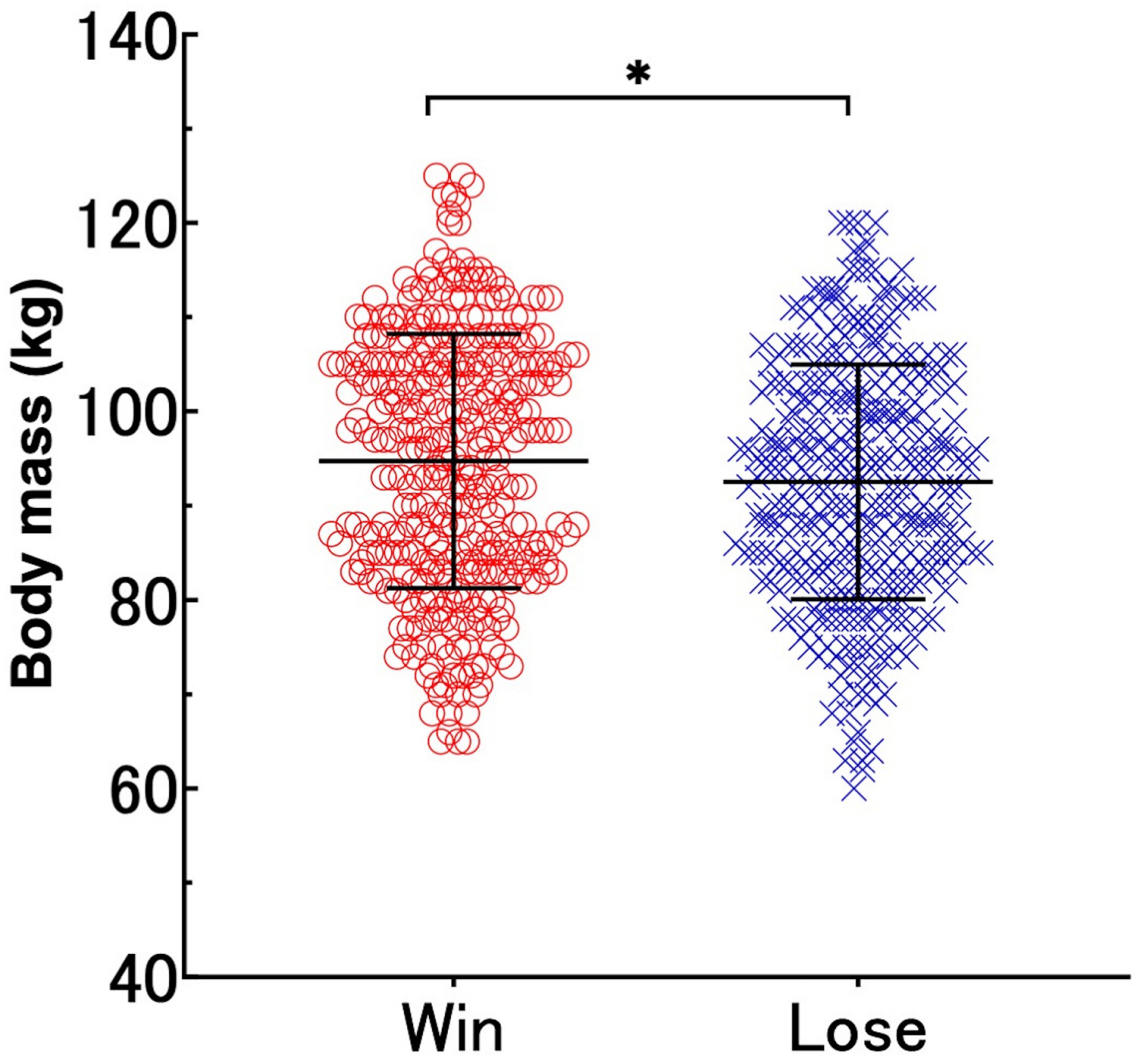
Distribution of body mass in relation to match outcomes (win vs. lose). Distribution of body mass (kg) for the matches resulting in either a win (left, red circles) or a loss (right, blue crosses). Each point represents an individual data point, with the horizontal lines indicating the mean and standard deviation for each group. Asterisk (*) denotes a statistically significant difference (*p* < 0.05).

**Figure 3:**
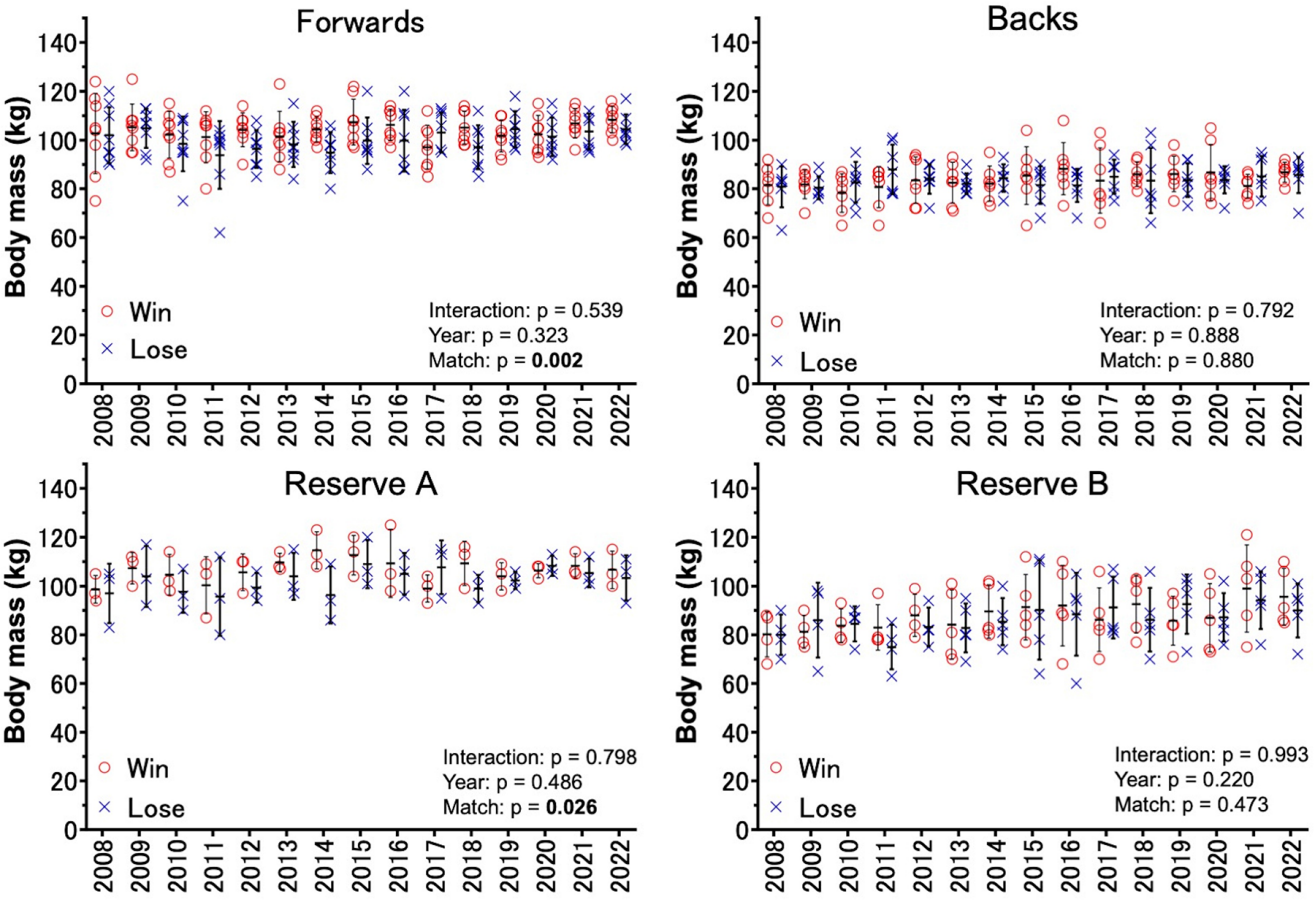
Comparison of body mass by position and match outcome over time (2008–2022). Forwards (top left), backs (top right), reserve **A** (bottom left), and reserve **B** (bottom right) from 2008 to 2022. The red circles (○) represent body mass in winning teams, and the blue crosses (✕) represent body mass in losing teams. The error bars indicate the standard deviations for each year.

**Figure 4:**
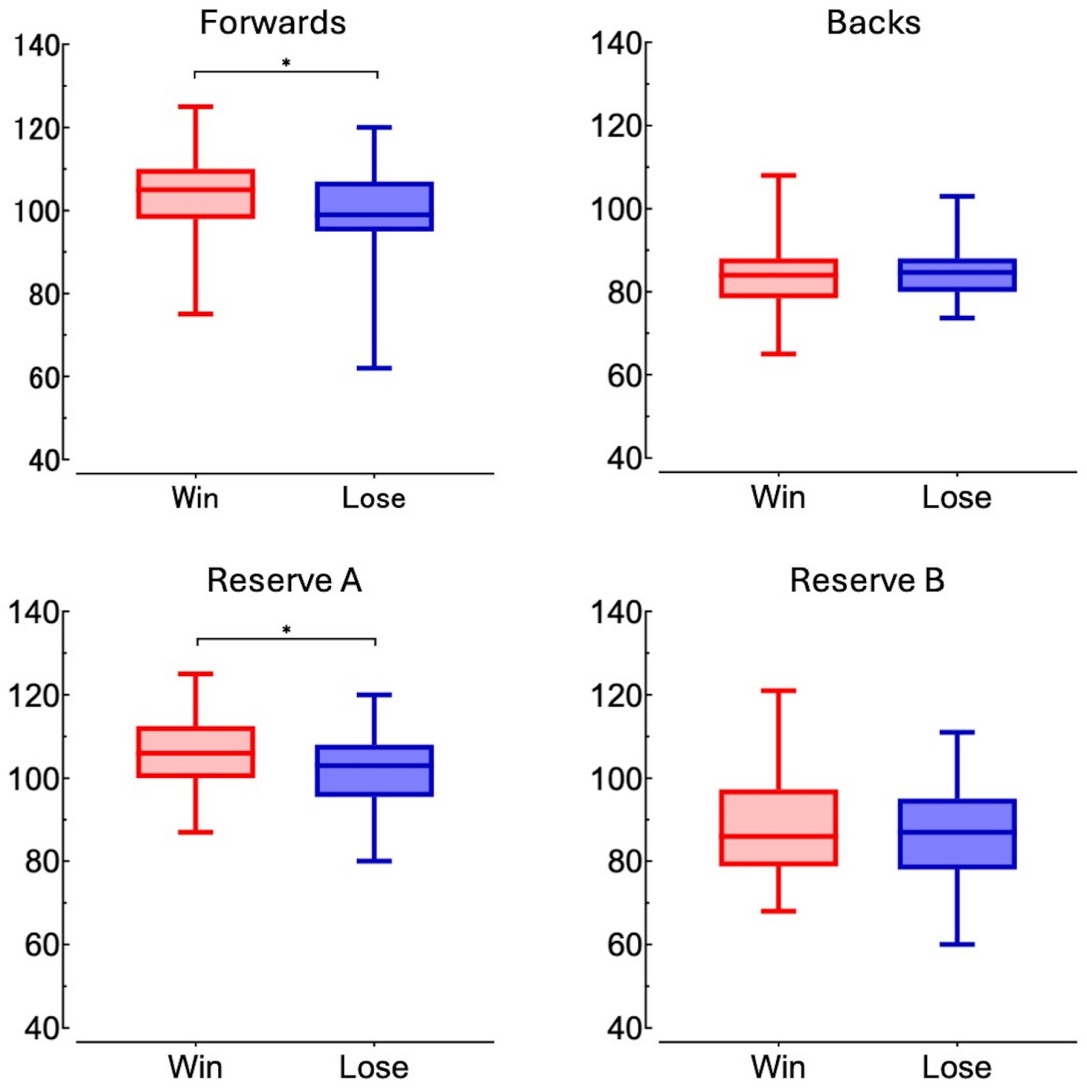
Distribution of body mass by position and match outcome (win vs. lose). Distribution of body mass (kg) for each player position (forwards, backs, reserve **A**, and reserve **B**) using box plots. The asterisk (*) denotes a significant difference (*p* < 0.05).

Supplementary Table S1 compares the characteristics of the winning and losing teams in the final match of university tournaments. No interaction was observed for body mass in matches over the 15 years for all registered members [interaction (year × match) was F = 0.616, *p* = 0.853, partial *η*^2^ = 0.013; Figure 1]. There were no significant main effects of year (F = 1.169, *p* = 0.295, partial *η*^2^ = 0.025; Figure 1), whereas a difference was observed in the main effects of the match outcome (F = 4.841, *p* = 0.028, partial *η*^2^ = 0.007; Figures 1, 2). The results revealed significant differences in body mass between the winning and loser teams, with the winners being 2.2 kg heavier on average (*p* = 0.027, d = 0.17; Figure 2). No interaction was observed for body mass in any of the four player designations (Figure 3): forwards (F = 0.918, *p* = 0.539, partial *η*^2^ = 0.058), backs (F = 0.679, *p* = 0.792, partial *η*^2^ = 0.050), reserve A (F = 0.665, *p* = 0.798, partial *η*^2^ = 0.134), and reserve B (F = 0.299, *p* = 0.993, partial *η*^2^ = 0.037). However, a marked difference in body mass was detected in the main effects of match in the forward (F = 9.452, *p* = 0.002, partial *η*^2^ = 0.043) and reserve A groups (F = 5.164, *p* = 0.026, partial *η*^2^ = 0.079) (Figure 3). The body mass of the winning team exceeded that of the losing team by 3.6 kg in the forward position (*p* = 0.002, d = 0.40) and by 4.2 kg in the reserve A position (*p* = 0.022, d = 0.49) (Figure 4). The body mass of players in positions #4 and #6 of the forward and player #16 in the reserve A group were greater on the winning team (Supplementary Table S2).

## Discussion

Using registration data from 680 players across 30 teams who participated in finals over the past 15 years, we analyzed the relationship between body mass and match outcomes. Although no statistical interactions were inferred regarding the finals over the 15 years, the winning teams were consistently heavier than the losing teams when considering the entire squad (Figures 1, 2). Specifically, in the forward and reserve A positions, heavier players were more likely to be on the winning team, highlighting the importance of body mass in forward positions (Figures 3, 4). According to Hill et al. (4), the mean body mass of international rugby players has increased substantially since 1995, with an increase of 24.3% from 84.8 ± 8.2 kg in 1955 to 105.4 ± 12.1 kg in 2015. However, our results between 2008 and 2022 showed no marked increase in the body mass of top university players. A national health and nutrition survey reported a 5 kg increase in the average weight of 20-year-old males from 2008 to 2012 (21), but the absence of such an increase among top university players may suggest that their primary focus is on increasing muscle mass and fat-free mass, making overall weight gain more challenging. A 0.5 kg increase in the body mass of top rugby players between 1999 and 2018 indicated that 0.8 kg of that increase was attributed to fat-free mass (22). Bevan et al. (22) reported that the mean body mass of top young rugby players in the same age group was 104 kg, suggesting that even the top players in Japanese university rugby are still physically and technically growing. This finding highlights the importance of adequate and efficient nutrition and training support.

One of the key findings of this study was that the winning team's forwards and reserve A, who are substitutes for forwards, were heavier, which is consistent with studies showing that the forwards of Rugby World Cup-winning teams are heavier than their opponents (23). The larger size of the forwards influences scrum strength and power, with weight, mesomorphy, maximum anaerobic power, and hip angle during the scrum accounting for 45% of the variance (11). Heavier players may also have an advantage in contact situations such as rucks and mauls, contributing to a team's success. At lower levels of competition, the weight difference between forwards and backs is not as pronounced, suggesting that the specificity of roles at the elite level is more clearly defined, which may reflect the high level of competition in university championship finals (6). In recent years, increased mobility, lower body fat, and greater muscle mass among forwards have also become relevant (6, 22). Therefore, in addition to physical body mass, increasing muscle mass to lower the center of gravity and enhance stability is crucial. Factors such as technique, speed, and tactics are intrinsically linked and may lead to different results at the professional level. Whereas the body mass of forwards has steadily increased over the past 60 years, that of backs has shown little increase since 1995 (4). Although heavier weights can improve impact force in tackles and the ability to push back opponents, excessive weight gain may reduce agility and endurance (24). Thus, the current study provides reasonable evidence that the optimal body mass for a given position and individual characteristics is critical for success. An analysis of professional rugby union players in England over a 10-year period from 2002 to 2011 revealed that only fly halves (#10) and back row (#6–#8) players experienced an increase in body mass (25). In the present study, no annual changes in body mass were observed over the 15-year period for either forwards or backs (Figure 3); the winning team have heavier players in the back five positions as forward players (#4 and #6) (Supplementary Table S2). This finding implies that body mass in these positions and roles may be a critical factor in determining match outcomes. Nevertheless, this positional difference may be influenced by variations in the developmental phases and strategies between professional and university-level players, which remains unclear in this investigation.

This study had several limitations. First, the data were derived exclusively from university rugby championship finals, which are potentially not representative of broader trends in university rugby or other competitive levels. Second, while body mass was analyzed, other components of body composition, such as muscle mass, fat-free mass, which could provide more detailed insights into player performance, were not measured directly. Third, the study did not account for game-related factors such as weather conditions, match strategies, or player fatigue, which may influence match outcomes. Fourth, the analysis focused solely on winning and losing teams, without considering other performance indicators. Finally, the use of registration data may introduce potential inaccuracies, as player weights may not have been consistently measured or updated throughout the season. Future studies should address these limitations by incorporating more comprehensive physical assessments such as body composition (fat-free mass, muscle mass, and bone mass), broader datasets, and game-related variables, to provide a deeper understanding of the relationship between body mass and match outcomes.

## Conclusions

Although no significant increase in body mass was observed among university players participating in the university rugby championship finals between 2008 and 2022, the winning teams comprised players with higher body mass. These findings indicate the necessity for position-specific strategies in training and nutrition to optimize player performance and competitiveness. For instance, strength and conditioning programs could be customized to assist players in key positions to develop the optimal balance of muscle mass and fat-free mass required for their respective roles. The results of this study underscore the importance of body composition at the elite university level, suggesting the need for further investigation.

## Data Availability

The original contributions presented in the study are included in the article/Supplementary Material, further inquiries can be directed to the corresponding author.
