# Supplementary material for: Trends in body mass between finalist teams in the Japanese collegiate rugby union championship: a 15-year analysis
Source: Front Sports Act Living. 2025 Feb 28;7:1496093. doi: 10.3389/fspor.2025.1496093 (PMC11906344; doi:10.3389/fspor.2025.1496093)
Supplement: Supplementary file 1 [file Table1.docx]

| **Win** |  | **Age (years)** | | |  | **Height (cm)** | | |  | **Body mass (kg)** | | |  | **BMI (kg/m^2^)** | | |  | **N** |
| --- | --- | --- | --- | --- | --- | --- | --- | --- | --- | --- | --- | --- | --- | --- | --- | --- | --- | --- |
| 2008 |  | 21.0 | ± | 1.3 |  | 177.3 | ± | 7.3 |  | 91.4 | ± | 15.4 |  | 29.0 | ± | 4.3 |  | 22 |
| 2009 |  | 21.1 | ± | 1.2 |  | 178.7 | ± | 6.1 |  | 93.7 | ± | 14.3 |  | 29.3 | ± | 3.8 |  | 22 |
| 2010 |  | 21.1 | ± | 1.2 |  | 177.3 | ± | 7.5 |  | 91.6 | ± | 14.2 |  | 29.0 | ± | 3.3 |  | 22 |
| 2011 |  | 21.3 | ± | 0.9 |  | 179.1 | ± | 8.1**^＊^** | | 91.3 | ± | 13.5 |  | 28.4 | ± | 3.2 |  | 22 |
| 2012 |  | 20.7 | ± | 1.2 |  | 178.8 | ± | 7.4 |  | 94.9 | ± | 12.7 |  | 29.6 | ± | 3.3 |  | 22 |
| 2013 |  | 20.6 | ± | 1.0**^＊^** | | 180.3 | ± | 6.8 |  | 93.0 | ± | 14.4 |  | 28.6 | ± | 3.7 |  | 23 |
| 2014 |  | 21.0 | ± | 0.9 |  | 180.4 | ± | 7.0 |  | 95.7 | ± | 13.9 |  | 29.3 | ± | 3.3 |  | 23 |
| 2015 |  | 21.0 | ± | 1.0 |  | 179.6 | ± | 6.7 |  | 97.9 | ± | 15.0 |  | 30.3 | ± | 3.8 |  | 23 |
| 2016 |  | 21.2 | ± | 1.1 |  | 180.3 | ± | 7.8 |  | 98.1 | ± | 13.9 |  | 30.1 | ± | 3.2 |  | 23 |
| 2017 |  | 20.9 | ± | 0.9 |  | 175.4 | ± | 6.4 |  | 90.8 | ± | 12.4 |  | 29.4 | ± | 3.0 |  | 23 |
| 2018 |  | 21.0 | ± | 0.9**^＊^** | | 179.5 | ± | 6.0 |  | 97.1 | ± | 11.9 |  | 30.1 | ± | 3.1 |  | 23 |
| 2019 |  | 20.7 | ± | 1.1 |  | 178.2 | ± | 5.6 |  | 93.8 | ± | 10.9 |  | 29.5 | ± | 2.8 |  | 23 |
| 2020 |  | 21.0 | ± | 1.2 |  | 176.1 | ± | 5.5 |  | 94.8 | ± | 12.9 |  | 30.5 | ± | 3.3 |  | 23 |
| 2021 |  | 20.9 | ± | 1.3 |  | 177.7 | ± | 7.0 |  | 97.5 | ± | 14.6 |  | 30.9 | ± | 4.0 |  | 23 |
| 2022 |  | 20.9 | ± | 0.8 |  | 178.5 | ± | 6.2 |  | 98.8 | ± | 11.5 |  | 31.0 | ± | 3.5 |  | 23 |
| **All** |  | **21.0** | **±** | **1.1** |  | **178.5** | **±** | **6.8** |  | **94.7** | **±** | **13.5^＊^** | | **29.7** | **±** | **3.5** |  | **340** |
|  |  |  |  |  |  |  |  |  |  |  |  |  |  |  |  |  |  |  |
| **Lose** |  | **Age (years)** | | |  | **Height (cm)** | | |  | **Body mass (kg)** | | |  | **BMI (kg/m^2^)** | | |  | **N** |
| 2008 |  | 21.1 | ± | 1.2 |  | 178.3 | ± | 7.1 |  | 90.6 | ± | 14.0 |  | 28.4 | ± | 3.8 |  | 22 |
| 2009 |  | 21.0 | ± | 0.8 |  | 178.6 | ± | 6.9 |  | 93.6 | ± | 14.5 |  | 29.2 | ± | 3.6 |  | 22 |
| 2010 |  | 21.1 | ± | 1.2 |  | 178.0 | ± | 6.1 |  | 90.8 | ± | 11.6 |  | 28.6 | ± | 3.0 |  | 22 |
| 2011 |  | 21.2 | ± | 0.8 |  | 174.6 | ± | 4.7 |  | 88.8 | ± | 13.6 |  | 29.1 | ± | 4.0 |  | 22 |
| 2012 |  | 20.9 | ± | 1.3 |  | 178.8 | ± | 5.4 |  | 90.5 | ± | 9.6 |  | 28.3 | ± | 2.9 |  | 22 |
| 2013 |  | 21.3 | ± | 1.0 |  | 176.8 | ± | 5.8 |  | 90.7 | ± | 11.9 |  | 29.0 | ± | 3.3 |  | 23 |
| 2014 |  | 21.1 | ± | 1.3 |  | 177.9 | ± | 5.7 |  | 89.8 | ± | 9.4 |  | 28.4 | ± | 2.9 |  | 23 |
| 2015 |  | 21.1 | ± | 0.9 |  | 177.1 | ± | 6.6 |  | 93.4 | ± | 15.0 |  | 29.6 | ± | 3.7 |  | 23 |
| 2016 |  | 21.1 | ± | 0.9 |  | 177.5 | ± | 6.2 |  | 92.4 | ± | 14.4 |  | 29.2 | ± | 3.5 |  | 23 |
| 2017 |  | 20.7 | ± | 1.1 |  | 178.6 | ± | 5.3 |  | 95.6 | ± | 12.5 |  | 29.9 | ± | 3.1 |  | 23 |
| 2018 |  | 20.9 | ± | 0.9 |  | 175.4 | ± | 6.4 |  | 90.8 | ± | 12.4 |  | 29.4 | ± | 3.0 |  | 23 |
| 2019 |  | 20.9 | ± | 1.1 |  | 179.2 | ± | 6.4 |  | 95.3 | ± | 11.9 |  | 29.6 | ± | 3.1 |  | 23 |
| 2020 |  | 21.0 | ± | 1.4 |  | 177.9 | ± | 5.1 |  | 93.8 | ± | 11.8 |  | 29.6 | ± | 3.3 |  | 23 |
| 2021 |  | 20.9 | ± | 1.0 |  | 179.7 | ± | 7.4 |  | 96.1 | ± | 11.6 |  | 29.7 | ± | 2.8 |  | 23 |
| 2022 |  | 20.8 | ± | 1.1 |  | 176.8 | ± | 5.8 |  | 95.5 | ± | 11.5 |  | 30.5 | ± | 3.5 |  | 23 |
| **All** |  | **21.0** | **±** | **1.1** |  | **177.7** | **±** | **6.1** |  | **92.5** | **±** | **12.4** |  | **29.3** | **±** | **3.3** |  | **340** |

**Table S1 Characteristics and their comparisons between winner and loser teams**

^＊^*P* <0.05 vs. loser within year

**Table S2 Comparison between winner and loser teams in each position**

|  | **Registration** |  | **Win** | | | |  | **Lose** | | | |  |  |
| --- | --- | --- | --- | --- | --- | --- | --- | --- | --- | --- | --- | --- | --- |
|  | **Number** |  | **Mean** |  | **SD** | **N** |  | **Mean** |  | **SD** | **N** |  | **P value** |
| **Forwards** | 1 |  | 107.3 | ± | 7.0 | 15 |  | 106.5 | ± | 7.3 | 15 |  | 0.743 |
|  | 2 |  | 101.7 | ± | 3.5 | 15 |  | 98.9 | ± | 5.5 | 15 |  | 0.108 |
|  | 3 |  | 115.1 | ± | 5.9 | 15 |  | 111.8 | ± | 6.6 | 15 |  | 0.155 |
|  | 4 |  | 105.3 | ± | 5.7 | 15 |  | 98.5 | ± | 5.6 | 15 |  | **0.003** |
|  | 5 |  | 106.7 | ± | 5.5 | 15 |  | 103.7 | ± | 5.6 | 15 |  | 0.141 |
|  | 6 |  | 100.9 | ± | 10.4 | 15 |  | 89.7 | ± | 10.1 | 15 |  | **0.006** |
|  | 7 |  | 92.5 | ± | 6.3 | 15 |  | 92.1 | ± | 6.7 | 15 |  | 0.868 |
|  | 8 |  | 100.7 | ± | 4.9 | 15 |  | 100.1 | ± | 6.8 | 15 |  | 0.783 |
| **Backs** | 9 |  | 71.9 | ± | 5.3 | 15 |  | 73.2 | ± | 5.9 | 15 |  | 0.517 |
|  | 10 |  | 85.4 | ± | 5.2 | 15 |  | 85.3 | ± | 6.3 | 15 |  | 0.975 |
|  | 11 |  | 82.8 | ± | 8.1 | 15 |  | 82.4 | ± | 4.9 | 15 |  | 0.871 |
|  | 12 |  | 87.1 | ± | 6.2 | 15 |  | 87.4 | ± | 7.1 | 15 |  | 0.914 |
|  | 13 |  | 90.3 | ± | 8.3 | 15 |  | 88.1 | ± | 6.8 | 15 |  | 0.436 |
|  | 14 |  | 83.7 | ± | 8.8 | 15 |  | 83.8 | ± | 5.9 | 15 |  | 0.961 |
|  | 15 |  | 84.4 | ± | 5.0 | 15 |  | 84.1 | ± | 5.1 | 15 |  | 0.885 |
| **Reserve A** | 16 |  | 104.6 | ± | 7.3 | 15 |  | 97.5 | ± | 7.9 | 15 |  | **0.016** |
|  | 17 |  | 107.4 | ± | 4.5 | 15 |  | 104.5 | ± | 6.6 | 15 |  | 0.176 |
|  | 18 |  | 107.3 | ± | 10.7 | 15 |  | 104.8 | ± | 10.7 | 15 |  | 0.522 |
| **Reserve B** | 19 |  | 100.1 | ± | 7.5 | 15 |  | 98.7 | ± | 7.3 | 15 |  | 0.591 |
|  | 20 |  | 92.5 | ± | 14.8 | 15 |  | 85.5 | ± | 14.0 | 15 |  | 0.190 |
|  | 21 |  | 78.5 | ± | 7.5 | 15 |  | 75.7 | ± | 7.9 | 15 |  | 0.328 |
|  | 22 |  | 84.9 | ± | 6.0 | 15 |  | 86.5 | ± | 9.3 | 15 |  | 0.580 |
|  | 23 |  | 84.0 | ± | 10.2 | 10 |  | 88.0 | ± | 7.3 | 10 |  | 0.327 |

*P*-values indicate winners vs. losers within the same position number
